# Oocyte collection and outcome following oncologic treatment: a retrospective multicentre study

**DOI:** 10.1007/s00520-024-08586-0

**Published:** 2024-05-29

**Authors:** Marta J. Fernández-González, Anja Borgmann-Staudt, Clara González Llagostera, Elena Ceballos-Garcia, Judith Gebauer, Andreas Jantke, Anke Barnbrock, Heribert Kentenich, Stephanie Klco-Brosius, Laura Lotz, Magdalena Balcerek

**Affiliations:** 1 Department of Paediatric Oncology and Haematology, Charité-Universitätsmedizin Berlin, corporate member of Freie Universität Berlin, Humboldt-Universität zu Berlin, and Berlin Institute of Health, Augustenburger Platz 1, 13353 Berlin, Germany; 2 Hospital Dexeus, Barcelona, Spain; 3 Hospital Gregorio Marañón, Madrid, Spain; 4 University Hospital of Schleswig-Holstein, Lübeck, Germany; 5 Kinderwunschärzte Berlin, Zentrum für Kinderwunschbehandlung und Fertilitätsprotektion, Berlin, Germany; 6 University Hospital Frankfurt, Frankfurt, Germany; 7 Fertility Centre, Berlin, Germany; 8 University Women’s Hospital Erlangen, Erlangen, Germany; 9 Berlin Institute of Health (BIH), Berlin, Germany

**Keywords:** Cancer survivor, Female, Fertility preservation, Cryopreservation, Assisted reproductive technologies

## Abstract

**Purpose:**

This study assesses fertility treatment outcomes in female patients who had undergone successful oocyte retrieval following cancer therapy.

**Methods:**

Between January 2020 and December 2022, we collected fertility treatment data from six participating centres in Spain and Germany. All patients associated with this data had undergone successful oocyte retrieval following cancer treatment.

**Results:**

Women had most frequently been diagnosed with a haematological (41.9%), breast (22.6%) or gynaecological malignancy (12.9%); two thirds (67.7%) had previously received a chemotherapy, half a radiotherapy (53.3%) and 45.2% had undergone surgery. On average, 7 years (range 0–28) had passed between cancer treatment and first ovarian stimulation cycle. Forty-nine ovarian stimulation cycles had been conducted on these 31 women between 2004 and 2021 (mean age at first oocyte collection following treatment: 34.8 ± 5.7 years). On average, 7 oocytes were collected per cycle (range 0–26) and 11 were collected per patient (range 0–51). Out of the 190 oocytes collected for immediate use of artificial reproductive technique, 139 were fertilised at a rate of 73%. Live birth rate per fresh transfer was 45% (9/20); no births were reported following cryotransfer (0/10). Mean values of anti-Mullerian hormone (AMH) before stimulation declined with time since treatment; however, oocytes were successfully collected from four women with an AMH of <0.5 ng/ml, although no pregnancies were reported. Ten pregnancies were documented; 3 ended in miscarriage. Two twin and 5 single pregnancies resulted in nine live births. On average, children were carried to term.

**Conclusion:**

In this small cohort, oocytes were successfully collected after chemotherapy and radiotherapy, despite—in individual cases—low AMH values. Further studies are needed to enrich the database and ultimately provide appropriate counselling to female cancer patients regarding expectations and ART outcome following cancer therapy.

**Supplementary Information:**

The online version contains supplementary material available at 10.1007/s00520-024-08586-0.

## Introduction

Due to the gonadotoxic potential of certain cancer treatments, a significant proportion of female cancer survivors face the risk of premature ovarian insufficiency (POI) which may result in a reduced fertile window and infertility [1, 2]. Large studies have identified alkylating agents as well as pelvic/gonadal irradiation as main risk factors for gonadotoxicity in female cancer patients [3]. Fertility can be safeguarded via oocyte (unfertilised or fertilised) and/or ovarian tissue cryopreservation in adolescent and adult cancer patients. Where feasible, guidelines recommend the use of fertility preservation measures prior to cancer treatment [4–6]. These options, though, have limitations, which depend, among other factors, on cancer diagnose and treatment. Oocyte retrieval requires patients to undergo a hormonal stimulation cycle of usually 14 days. However, due to the life-threatening nature of oncologic diseases, a delay of treatment is not always feasible for all patients. Surgical collection of ovarian tissue should be combined with other procedures which require anaesthesia such as the implantation of a central venous catheter; yet, the logistics of ovarian tissue cryopreservation is often complex, as it requires careful interdisciplinary planning as only few centres are experienced in this procedure. Additionally, ovarian tissue collected prior to cancer treatment may be infiltrated with malignant cells, especially in patients with haematologic malignancies but, also in various solid tumours, posing a risk of relapse if ovarian tissue is auto-transplanted [7, 8]. Current experimental approaches to overcome this barrier include in vitro maturation (IVM) of early stage oocytes or artificial ovaries [9, 10]. Independent of whether or not fertility preservation measures were used prior to treatment, current guidelines recommend fertility surveillance and discussion of results and preservation options within the routine clinical follow-up after oncologic treatment [4–6]. Fertility monitoring following cancer treatment should include menstrual cycle information, which can be of value in detecting fertility irregularities, as well as hormone analyses, including anti-Mullerian hormone (AMH), a marker for ovarian reserve, and if indicated the antral follicle count (AFC) measured by ultrasound by a reproductive specialist.

Recent studies have focused on determining the efficacy of fertility therapies in female cancer patients who underwent oocyte collection prior to cancer treatment. On a somewhat larger scale, a publication by Dolmans et al. assessed cryopreservation of ovarian tissue before and after the start of therapy [9]. There is, however, only limited data on the outcome of the use of oocytes collected after cancer therapy. We therefore aim to describe fertility treatment outcomes in female patients for whom oocytes were successfully preserved following cancer therapy.

## Material and methods

Six centres in Spain and Germany participated in our retrospective study between January 2020 and December 2022. Data from female cancer patients treated between 2004 and 2021 were extracted from hospital case files. Fertility data, as well as cancer diagnosis and treatment information, were collected from those patients who had undergone successful oocyte retrieval following cancer treatment. In the absence of data availability from the participating centre, written informed consent was requested from former patients—either to obtain missing data from their oncologist or to self-report it in a short questionnaire. All study data was documented pseudonymised. Research was conducted in accordance with the Declaration of Helsinki. The ethics committees of Charité—Universitätsmedizin Berlin (EA4/158/19), as well as of participating centres, approved the study.

Data was included on all women for whom oocytes had successfully been collected following an oncologic treatment that involved chemotherapy and/or radiotherapy with or without surgery, independent of whether oocytes were cryopreserved for future utilisation, or whether they were directly used for artificial reproductive technologies (ART), such as intracytoplasmic sperm injection (ICSI) or in vitro fertilisation (IVF). Study inclusion was considered only for those patients who had documented cancer diagnosis information at a participating centre. A total of 66 women were identified to have undergone successful oocyte collection after initiation of cancer treatment during childhood, adolescence or adulthood. Overall, 31/66 of these women were included in our study; 16 were excluded due to unavailability of detailed treatment information and 19 had undergone treatment that did not include chemotherapy or radiotherapy. Patients were stratified according to time since end of oncologic treatment to first oocyte cycle (group 1 = 0–3 years, group 2 = 4–10 years, group 3 = ≥11 years). Cancer diagnoses were classified as haematological cancers (leukaemia or lymphoma), gynaecological cancers (ovarian, cervical and endometrial cancer), breast cancer and cancers in other locations.

We assessed patient characteristics and ovarian stimulation characteristics as well as outcome of fertility treatment with oocytes collected following chemotherapy and/or radiotherapy. Hormonal stimulation consisted of GnRH-agonist or GnRH-antagonist stimulation protocols with or without aromatase inhibitor. Ovarian stimulation characteristics included type of agent used for stimulation, mean hormone values at stimulation (follicle stimulating hormone (FSH), luteinising hormone (LH), estradiol, progesterone, AMH) and number of follicles >14 mm in size, assessed with transvaginal ultrasonography between the second and fourth day of menstrual cycle during hormonal stimulation. Fertility treatment outcomes included fertilisation rate (number of fertilised oocytes per oocyte inseminated), pregnancy rate (number of pregnancies with at least one gestational sac divided by the number of total cycles), miscarriage rate (number of spontaneous abortions divided by the number of total pregnancies) and live birth rate (live births divided by the number of total patients). We furthermore assessed newborn characteristics using WHO definitions to describe perinatal outcomes (gestational age, weight and length at birth) [11].

Data analysis was conducted using R, version 3.6.1. Descriptive analysis was conducted. All continuous variables are presented as means and standard deviations (SD) or median values and interquartile ranges (IQR). Power calculation was not performed as this was a retrospective study and the sample size derived from data directly obtained from different hospital databases.

## Results

### Patient characteristics

The 31 women identified to have undergone oocyte collection following a cancer treatment were on average 26.6 ± 8.9 years old at the time of their cancer diagnosis; five patients (16.1%) were younger than 18 years of age. On average, there was an interval of 7.1 years (range 0–28) between end of cancer treatment and the first oocyte retrieval. One patient had underwent successful oocyte collection following standard chemotherapy and before high-dose cancer treatment with stem cell transplantation. Almost half of the patients (45.2%) were older than 35 years at time of oocyte collection (mean age: 34.8 ± 5.7 years). Patients’ mean BMI before oocyte collection was 23.1 ± 3.6 kg/m^2^, the mean AMH was 1.7 ± 1.6 ng/ml and the mean AFC 11.2 ± 10.0. Patient characteristics are shown in Table 1.

**Table 1:** Patient characteristics

	Variables	MD	Time between end of cancer treatment and first oocyte collection							
			0–4 years^1^		5–10 years		>11 years		All	
	No of cancer patients—*n* (%)	0	13	41.9	10	32.3	8	25.8	31	100
	Mean interval in years (end of cancer treatment to 1st cycle [SD])	0	1.5 [1.2]	–	6.9 [1.5]	–	16.5 [5.9]	–	7.1 [6.8]	–
	Median interval in years (end of cancer treatment to 1st cycle [IQR])		2.0 [2.0]	–	7.0 [1.8]	–	14.5 [7]	–	6.0 [8.5]	–
Age	Mean age at 1st cancer treatment initiation in years [SD]	0	32.9 [6.7]	–	25.2 [4.7]	–	18.3. [8.8]	–	26.6 [8.9]	–
	Median age at 1st cancer treatment initiation in years [IQR]	0	33.0 [4.1]	–	26 [4.2]	–	19.3 [8.8]	–	28 [9.5]	–
	<18—*n* (%)	0	1	7.7	1	11.1	3	37.5	5	16.1
	≥18—*n* (%)		12	92.3	9	88.9	5	62.5	26	83.9
Cancer therapy	Year of 1st cancer treatment 1984–1999	0	0	0	0		5	62.5	5	16.1
	2000–2009		1	7.7	8	80	3	37.5	12	38.7
	2010–2020		12	92.3	2	20	0	0	14	45.2
	Chemotherapy (yes)—*n* (%)	0	7/13	53.9	8/10	80	6/8	75	21/31	67.7
	Radiotherapy (yes)—*n* (%)	1	8/13	61.5	4/9	44.4	4/8	50	16/30	53.3
	TBI	2	0	0	0	0	3	60	3/14	21.4
	Pelvic irradiation		0	0	1^2^	20	0	0	1/14	7.1
	Cranial		1	16.7	1^2^	20	1	40	2/14	14.3
	Others		5	83.3	3	60	0	0	8/14	57.2
	Surgery (yes)—*n* (%)	0	8/13	61.5	3/10	30	3/8	62.5	14/31	45.2
	Ovarian surgery		4/8	50	3/3	23.1	2/3	15.4	9/14	64.3
Cancer diagnosis	Haematological malignancy	0	5	38.5	4	40	4	50	13	41.9
	Gynaecological-Ca		2	15.4	1	10	1	12.5	4	12.9
	Breast-Ca		3	23.1	3	30	1	12.5	7	22.6
	Others		3	23.0	2	20	2	25	7	22.6
Fertility cycle	Mean age at 1st oocyte collection in years [SD]	0	34.9 [6.5]	–	33.6 [4.8]	–	36.1 [5.9]	–	34.8 [5.7]	–
	Median age at 1st oocyte collection in years [IQR]	0	37.0 [4.2]	–	34.5 [2.7]	–	37.2 [8.1]	–	35.6 [5.1]	–
	<35—*n* (%)^3^	0	6/13	46.2	8/10	80	3/8	37.5	17/31	54.8
	≥35—*n* (%)^3^		7/13	53.8	2/10	20	5/8	62.5	14/31	45.2
	Year of 1st ART cycle 2004–2014	0	4	30.8	4	40	4	50	12	38.7
	2015–2018		4	30.8	4	40	2	25	10	32.3
	2019–2021		5	38.4	2	20	2	25	9	29.0
	Mean BMI at 1st stimulation in kg/m^2^ [SD]	3	23.1 [3.8]	–	21.8 [7.2]	–	21 [9.2]	–	23.1 [3.6]	–
	BMI at 1st stimulation—low weight (<18.5)	3	2	18.2	0	0	0	0	2	7.1
	Normal weight (18.5–24.9)		7	63.6	8	88.9	4	50	19	67.9
	Overweight (25–29.9)		2	18.2	1	11.11	4	50	7	27

*MD* missing data, *SD* standard deviation, *IQR* interquartile range, *HSCT* haematopoietic stem cell transplantation, *TBI* total body irradiation

^1^One patient underwent oocyte collection following standard chemotherapy and before high-dose cancer treatment with stem cell transplantation; all others underwent collection after the end of the oncological treatment. ^2^Both sites were in the same patient; for the total value, the pelvic is counted. ^3^Cobo et al. [12]

### Ovarian stimulation and fertility cycle outcomes

Overall, 49 ovarian stimulation cycles were documented for 31 women. The reasons for oocyte collection following cancer treatment included medical egg freezing and low ovarian reserve, as well as previous miscarriages or insemination failure. More than half (19/31, 61.3%) had only undergone one stimulation cycle. The majority of stimulations were conducted using a GnRH antagonist (Table 1, supplemental material). On average, 6.9 oocytes were collected per cycle (range 0–26) and 11 per patient. In patients younger than 35 years at time of oocyte retrieval, the mean number of oocytes collected did not decrease as time from end of treatment increased (group 1 = 7.2 oocytes, group 2 = 7.8 oocytes, group 3 = 8 oocytes). For patients 35 years and older, the mean number of oocytes collected decreased as the interval between end of cancer treatment and first oocyte collection increased (group 1 = 8.6 oocytes, group 2 = 7.9 oocytes, group 3 = 7.5 oocytes). Generally, as time since treatment increased, rate of mature (metaphase II, MII) oocytes decreased (group 1 = 79.2%, group 2 = 71.1%, group 3 = 65.2%).

Twenty-five women had used their fresh oocytes (*n* = 190) retrieved from 31/49 cycles for IVF or ICSI. These oocytes were fertilised at a rate of 73.2% (139/190) and led to live births for 7 out of 25 women (36%). An additional 11 cycles required cancellation and from 7/49 cycles, oocytes were cryopreserved for future ART. Further information regarding ovarian stimulation and fertility cycle outcomes is presented in Table 2.

**Table 2:** Fertility outcomes

Variables	MD	Time between end of cancer treatment and oocyte collection							
		0–4 years		5–10 years		>11 years		All	
ART cycles—*n* (%)	0	19	38.8	20	40.8	10	20.4	49	100
Medical oocyte freezing (MOF)	0	4	25	2	10.5	1	9.1	7	14.3
IVF—*n* (%)		3	18.8	1	5.3	1^3^	9.1	5	10.2
ICSI—*n* (%)		12	46.2	7	26.9	7^3^	26.9	26	53.1
No fertilisation^1^		0	0	9	47.4	2	18.2	11	22.4
Number of cycles = 1	0	8	61.5	5	50.	6	75	19	61.3
Number of cycles = 2–3		5	38.5	4	40	2	25	11	35.5
Number of cycles ≥ 4		0	0	1	10	0	0	1	3.2
Cycle cancellation^2^		0	0	4	80	1	20	5	100
No of total oocytes/cycle	0	7.8 (148/19)	–	6.2 (123/20)	–	6.9 (69/10)	–	6.9 (340/49)	–
No of total oocytes/patient		11.4 (148/13)		12.3 (123/10)		8.6 (69/8)		11.0 (123/31)	
Oocytes maturation (MII oocytes/all collected)	5	100/126	79.2	79/111	71.1	43/66	65.2	222/303	73.3
No of cryopreserved oocytes/total oocytes retrieved	0	33/135	24.4	5/123	4.1	3/69	4.4	41/327	12.5
No of cryopreserved embryos/total fertilised oocytes	0	14/40	35	6/61	9.8	6/29	20.7	26/130	20
Fertilisation rate	1	49/68	72.1	61/79	77.2	29/43	67.4	139/190	73.2
Pregnancy rate per transfer	0	6/17	35.3	3/7	42.9	1/6	16.7	10/30	33.3
Pregnancy rate per fresh transfer		5/10	50	2/5	40	1/5	20	8/20	40
Miscarriage rate (miscarriage/total pregnancy)	0	1/6	16.7	2/3	66.7	0/1	0	3/10	30.2
Miscarriage rate (miscarriage/total cryotransfer)	0	1/7	14.3	1/2	50	0/1	0	2/10	20
Total offspring born after ART per transfer cycle	0	5/17	29.4	2/7	28.6	2/6	33.3	9/30	30.0
Live birth rate (without MOF)	0	5/10	50	2/8	25	2/7	28.6	9/25	36
Live birth rate following fresh transfer		5/10	50	2/5	40	2/5	40	9/20	45
Live birth rate following cryotransfer	0	0/7	0	0/2	0	0/1	0	0/10	0
Mean number of ET [SD]	0	1.3 [1.7]	–	1 [0.9]	–	0.9 [0.4]	–	1.0 [1.2]	–

*MD* missing data, *SD* standard deviation, *ART* assisted reproductive technology, *ICSI* intracytoplasmic sperm injection, *IVF* in vitro fertilisation, *ET* embryo transfer, *hCG* human chorionic gonadotropin, *MOF* medical oocyte freezing

^1^Cycle cancellation/no oocytes retrieved/oocyte degenerated/embryos no fertilised; ^2^low responders, failure of hormone stimulation; ^3^one cycle was mix IVF and ICSI

According to the AMH value prior to stimulation, mean number of oocytes retrieved per cycle was highest in the group of women with an AMH >1.1 ng/ml (on average 13.0, Table 3). Four women had an AMH of <0.5 ng/ml prior to oocyte collection. In each of these 4 women, only a single stimulation cycle was carried out and led to retrieval of an average of 3.5 oocytes per cycle (range 1–9, Table 2). Single transfer was attempted for two of these women (breast cancer), one had degenerated oocytes (ovarian cancer) and another had her retrieved oocytes cryopreserved (brain cancer). No pregnancies were recorded for any of these four patients.

**Table 3:** Patients results depending on AMH levels and time between end of cancer treatment and oocyte collection

AMH (ng/ml)		Time between end of cancer treatment and oocyte collection							
		0–4 years		5–10 years		>11 years		All	
AMH > 1.1^1^	Number of patients	4	44.4	4	44.4	1	11.1	9	100
	Number of cycles	5	45.5	5	45.5	1	9.1	11	100
	Mean age at oocyte collection [SD]	38.1 [4.5]	–	35.6 [3.1]	–	38.00 [–]	–	36.6 [3.7]	–
	Mean AMH [SD]	4.1 [1.4]	–	2.1 [1.2]	–	1.20 [–]	–	2.9 [1.6]	–
	Mean oocytes retrieved [SD]	15.6 [8.1]	–	13.8 [11.2]	–	2 [–]	–	13.0 [9.9]	–
	Mean total follicles ≥14 mm	2.0 [1.4]	–	7.6 [7.7]	–	13.00 [–]	–	4.7 [6.0]	–
	Pregnancy rate^2^	1/5	20	1/5	20	0/1	0	2/11	18.2
	Miscarriages	1/5^4(1)^	20	1/5^4(2)^	20	0/1	0	2/11	18.2
	Live birth rate^2^	2/5	40	0/5	0	0/1	0	2/11	18.2
AMH 0.5–1.1^1^	Number of patients	2	40	3	60	0	–	5	100
	Number of cycles	3	37.5	5	62.5	–	–	8	100
	Mean age at oocyte collection [SD]	37.7 [0.5]	–	33.0 [6.5]	–	–	–	34.8 [5.5]	–
	Mean AMH [SD]	0.8 [0.1]	–	0.7 [0.2]	–	–	–	0.6 [0.3]	–
	Mean oocytes retrieved [SD]	1.7 [1.5]	–	1.6 [1.1]	–	–	–	1.6 [1.2]	–
	Mean total follicles ≥14 mm	4.0 [2.8]	–	9.0 [7.5]	–	–	–	7.1 [6.4]	–
	Pregnancy rate^3^	0/3	0	0/5	0	–	–	0/8	0
	Miscarriages	0/3	0	1/5^4(3)^	100	–	–	1/8	12.5
	Live birth rate^3^	0/3	0	0/5	0	–	–	0/8	0
AMH < 0.5^1^	Number of patients	0	–	2	50	2	50	4	100
	Number of cycles	–	–	2	50	2	50	4	100
	Mean age at oocyte collection [SD]	–	–	34.6 [1.7]	–	34.5 [11.8]	–	34.5 [6.9]	–
	Mean AMH [SD]	–	–	0.1 [0.0]	–	0.3 [0.0]	–	0.2 [0.1]	–
	Mean oocytes retrieved [SD]	–	–	1.0 [0.0]	–	6.0 [4.2]	–	3.5 [3.8]	–
	Mean total follicles ≥14 mm	–	–	3.5 [0.7]	–	18.0 [7.1]	–	10.8 [9.3]	–
	Pregnancy rate^2^	–	–	0/2	0	0/2	0	0/4	0
	Miscarriages	0	–	0	–	0	–	0	–
	Live birth rate^2^	–	–	0/2	0	0/2	0	0/4	0

*SD* standard deviation

^1^Ferrareti et al. 2011; ^2^one patient did not try; ^3^two patients did not try; ^4(1)^exact week unknown; ^4(2)^after 9 weeks; ^4(3)^after 12 weeks

### Perinatal outcomes following the use of oocytes collected after cancer treatment

Overall, 10 pregnancies were documented, of which 3 were miscarriages, resulting in 9 live births including two twin births. On average, children were carried to term (mean 38.6 weeks gestation, range 36–40) and with an average birth weight of 2833.8 g (range 2300–3250). Only 2 out of 8 children were born spontaneously; the other 6 were delivered by caesarean section. Table 4 presents perinatal outcomes of new-borns whose mothers had undergone ART using oocytes collected following cancer treatment.

**Table 4:** Perinatal newborn characteristics

Variables	MD	Time between end of cancer treatment and oocyte collection							
		0–4 years		5–10 years		>11 years		All	
Number of multiple sibling births		0/5	0	1/1	100	1/1	100	2/7	286
Mean gestation time (in weeks) [SD]		38.8 [1.0]	–	40	–	36	–	38.6 [1.4]	–
Preterm birth (<37 weeks gestation)^1^		0/5	0	0/1	0	1/1	100	1/7	14.6
Mean birth weight (g [SD])		2907.5 [309.5]	–	2650 [950.0]	–	2515 [21.2]	–	2833.8 [349.4]	–
Low birth weight (<2500 g)^1^		0/6	0	½	50	0/2	0	1/8	12.5
Birth mode: natural		2	33.3	0	0	0	0	2/8	25
Caesarean section		4	66.7	1	100	1	100	6/8	75

*MD* missing data, *SD* standard deviation

^1^World Health Organisation definitions were employed (https://www.who.int) [11]

## Discussion

Fertility problems as a result of chemotherapy and/or radiotherapy affects one third of all cancer patients; this risk increases after haematologic stem cell transplantation (HSCT) [1, 2]. Guidelines recommend that patients are offered the use of fertility preservation prior to a gonadotoxic treatment [2, 13] as fertility may become impaired immediately or soon after (e.g. following HSCT after a mean of 2.5 years [14]). Awareness of the risk for fertility impairment and of fertility preserving measures has increased in the oncologic setting in recent years and has also led to insurance coverage of these procedures in some countries—however, insurance coverage is potentially limited to adult patients in some cases. Further limitations related to patient age, diagnosis and urgency of treatment as well as lack of accessibility of procedures and necessary infrastructures may additionally hinder fertility preservation prior to cancer treatment. Survivors of childhood and/or adolescent cancer may be most affected; low rates of fertility preservation prior to oncologic treatment have been reported in this population [1] and the interval from end of treatment to family planning may be most significant. Without proper surveillance, drastically reduced fertile windows could be missed. Accordingly, former childhood cancer patients may enter menopause prematurely despite potentially initial unsuspicious pubertal development or menstrual cycles following cancer treatments. In our cohort, patients treated for early childhood cancer were older when they began fertility treatment, which may additionally decrease chances for success. Detection of the reproductive window is essential as post-treatment oocyte collection may pose the only option of fertility protection especially in patients whose oncologic treatment required an urgent start or prepubertal children for who availability of fertility preservation may be limited.

Currently, little data is available on the outcome of oocyte retrieval following cancer treatment. We addressed this important topic in this retrospective multicentre study. Forty-nine ovarian stimulation cycles conducted in 31 women from participating centres. This low number of identified patients underlines the importance of focused patient counselling regarding fertility treatment options, as fertility problems following gonadotoxic treatment are not uncommon [1, 2]. Current literature also only anecdotally report of patients who had undergone oocyte collection following cancer treatment, including case reports on successful live births—most commonly among women who had suffered from breast and gynaecological cancers [15–20]. Further reports include the births of a healthy infant from oocytes collected following intense cancer treatment (including HSCT) of a women with acute myeloid leukaemia [21] and of a child from oocytes successfully harvested from a patient following cranial tumour radiotherapy and chemotherapy [22]. This underscores the growing necessity for future studies aimed at creating a broader knowledge base that will allow individualised patient counselling, especially those who seek fertility assistance following cancer treatment.

Hormone-induced ovulation is required to obtain sufficient numbers of oocytes. Ovarian response to ovulation induction was previously reported to be significantly diminished following cancer treatment [23]. In our cohort of patients who underwent stimulation following cancer treatment, high estradiol levels at time of human chorionic gonadotropin (hCG) administration, 36 h before oocyte collection, indicated a sufficient response to stimulation at all-time points after cancer treatment, even when initial AMH values were low. However, tumour and treatment-related factors seem to play a role for stimulation success: Ginsburg et al. reported a poorer response to gonadotropins in 15 women who underwent IVF after systemic cancer treatment compared to 56 women with locally treated cancers [24]. Data regarding stimulation and oocyte collection during chemotherapy is rare, as it is not recommended. Dolmans et al., who looked at ovarian stimulation conducted before the start of chemotherapy vs. following one to three courses of chemotherapy (4–14 weeks), concluded that the efficacy of IVF is dramatically reduced once chemotherapy begins or during chemotherapy [9]. In our post-cancer treatment cohort, an average of 7 oocytes were collected per cycle (range 0–26). This number did not decline with increasing time interval since treatment but decreased depending on patient age at stimulation (>36 years). In a study conducted by Chan et al., oocyte retrieval, once achieved, had similar outcomes in terms of oocyte yield and maturation rates compared to oocytes retrieved from patients prior to cancer treatment [23]. In our cohort, fertilisation and live birth rates following fresh oocyte were comparable to data from the German IVF register with fertilisation rates being somewhat lower and the live birth rate somewhat higher in our group [25]. Even so, the difference between the results obtained after fresh transfer and cryotransfer is noteworthy—no long-term pregnancies (>5 months) and live births were reported after 10 cryotransfers in our cohort. Some patients opted to cryopreserve oocytes (fertilised/unfertilised) for future ART and we can therefore not (yet) report on pregnancies/live birth rates for these patients. The miscarriage rate of 30% may be attributed to the ART procedure itself. Previous reports have generally shown similar rates of miscarriages in survivors and the general population [26], yet, in the subgroup of patients who required radiation that included the pelvis, uterine damage may increase this risk [27].

Before stimulation, AMH is assessed to estimate ovarian reserve. The magnitude of fertility impairment and the potential of recovery following cancer treatment is related to the extent of alkylating agent exposure and baseline ovarian reserve [28]. Individual variations in ovarian recovery limit the predictive value of AMH. As time interval since cancer treatment increased, AMH values declined in our cohort; the potential of recovery being low, especially in patients aged >40 years [29]. Our cohort also included four women who had AMH values <0.5 ng/ml, which is indicative of fertility impairment/potential premature ovarian failure; yet, oocytes were successfully collected following cancer treatment, although no pregnancies were achieved. Even though oocyte collection seems to be possible at low AMH levels, oocyte quality may proof to be the true predictor of successful fertility assistance.

### Study limitations

In this small heterogenous cohort of cancer survivors, we can only present descriptive data. However, as oocyte retrieval was successful following cancer treatment, this data adds valuable knowledge to patient counselling and also addresses gaps in current literature regarding fertility preservation. In 2023, FertiTox, a platform for the gonadotoxicity of cancer patients was kicked-off in Germany, Austria and Switzerland. This network will centrally record diagnostic and therapy data with follow-ups of 1 and 5 years. In our cohort, sixteen patients could not be considered for inclusion due to missing oncologic data. The precise knowledge of cancer history, including cancer treatment, is essential for late effects monitoring in survivors and its documentation should therefore be standard practice for all—including non-oncological—clinics that treat survivors. Documentation of precise cancer stage, protocol and anticancer agents would also support a broadened knowledge base enabling more individualised counselling. While procedures such as stimulations follow protocols and are standardised, and values such as laboratory results of hormones are reliable, AFC measurement is investigator-dependent and its relevance is therefore not considered in this study.

## Conclusions

This small overview of patient data reveals that it is possible to retrieve viable oocytes from patients after chemotherapy and/or radiotherapy, even when AMH levels are low. Preferably, when fertility preservation does not occur prior to cancer treatment initiation, oocyte collection should be considered and/or integrated into regular cancer follow-ups; this could be especially beneficial for patients with a short fertile window. Further studies to enrich the database are needed to offer appropriate counselling of female cancer patients on the expected outcome of oocytes collected after cancer therapy. In particular, investigations that test the fertility of oocytes collected at low AMH values are crucial.

### Supplementary information

ESM 1 (DOCX 16 kb)

## Data Availability

The data underlying this article will be shared on reasonable request to the corresponding author.
