# Supplementary material for: Oocyte collection and outcome following oncologic treatment: a retrospective multicentre study
Source: Support Care Cancer. 2024 May 29;32(6):390. doi: 10.1007/s00520-024-08586-0 (PMC11133171; doi:10.1007/s00520-024-08586-0)
Supplement: Supplementary file 1 — (DOCX 16 kb) [file 520_2024_8586_MOESM1_ESM.docx]

**Table 1 (Supplemental material)**. 1^st^ Ovarian Stimulation and hormones.

| **Variables** |  | **Time between end of cancer treatment and oocyte collection** | | | | | | | |
| --- | --- | --- | --- | --- | --- | --- | --- | --- | --- |
|  | **MD** | **0 – 4 years** | | **5 – 10 years** | | **>11 years** | | **All** | |
| Ovarian stimulation protocol: GnRH antagonist | 7 | 12 | 70.6 | 13 | 86.7 | 9 | 90 | 34 | 80.9 |
| GnRH agonist long protocol |  | 5 | 29.4 | 0 | 0 | 1 | 10 | 6 | 14.3 |
| Aromatase inhibitor |  | 0 | 0 | 2¹ | 13.3 | 0 | 0 | 2 | 4.8 |
| Mean AMH (ng/ml) prior to stimulation [SD] | 11 | 2.6 [1.9] | ---- | 1.22 [1.2] | ---- | 0.6 [0.5] | ---- | 1.7 [1.6] | ---- |
| Mean number of oocytes collected per patient with <0.5 ng/ml AMH |  | ---- | ---- | 1 [0] | ---- | 6 [4.2] | ---- | 3.5 [3.8] | ---- |
| Mean FSH level (U/l) prior to stimulation [SD] | 6 | 8.3 [4.4] | ---- | 12.1 [9.1] | ---- | 9.5 [11.7] | ---- | 10.1 [8.0] | ---- |
| Mean LH level (U/l) prior to stimulation [SD] | 19 | 2.6 [2.1] | ---- | 6.1 [3.6] | ---- | 0.3 [----] | ---- | 3.6 [3.1] | ---- |
| Mean estradiol level (pg/l) at time of hCG [SD] | 1 | 1013.1 [1225.4] | ---- | 1253.7 [946.9] | ---- | 1729.4 [1545.0] |  | 1284.3 [1202.1] | ---- |
| Mean total AFC [SD] | 11 | 8.4 [4.8] | ---- | 9.6 [4.8] | ---- | 16.3 [16.3] | ---- | 11.2 [10.0] | ---- |
| Mean total follicles ≥14mm | 0 | 5.6 [7.0] | ---- | 6.5 [6.9] | ---- | 8.5 [5.7] | ---- | 6.7 [6.5] | ---- |

MD, missing data; SD, standard deviation; ART, assisted reproductive technology; ICSI, intracytoplasmic sperm injection; IVF, in vitro fertilisation; IUI-H, Homologous intrauterine insemination artificial insemination; AFC, antral follicle count; AMH, Anti-müllerian hormone; FSH, Follicle stimulating hormone; LH, luteinizing hormone; hCG, human chorionic gonadotropin

¹Combined with GnRH Antagonist
